# One-step synthesis of high quality kesterite Cu_2_ZnSnS_4_ nanocrystals – a hydrothermal approach

**DOI:** 10.3762/bjnano.5.51

**Published:** 2014-04-09

**Authors:** Vincent Tiing Tiong, John Bell, Hongxia Wang

**Affiliations:** 1 School of Chemistry, Physics and Mechanical Engineering, Science and Engineering Faculty, Queensland University of Technology, Brisbane, QLD 4001, Australia

**Keywords:** Cu_2_ZnSnS_4_ nanocrystals, formation mechanism, hydrothermal, thioglycolic acid

## Abstract

The present work demonstrates a systematic approach for the synthesis of pure kesterite-phase Cu_2_ZnSnS_4_ (CZTS) nanocrystals with a uniform size distribution by a one-step, thioglycolic acid (TGA)-assisted hydrothermal route. The formation mechanism and the role of TGA in the formation of CZTS compound were thoroughly studied. It has been found that TGA interacted with Cu^2+^ to form Cu^+^ at the initial reaction stage and controlled the crystal-growth of CZTS nanocrystals during the hydrothermal reaction. The consequence of the reduction of Cu^2+^ to Cu^+^ led to the formation Cu_2−_*_x_*S nuclei, which acted as the crystal framework for the formation of CZTS compound. CZTS was formed by the diffusion of Zn^2+^ and Sn^4+^ cations to the lattice of Cu_2−_*_x_*S during the hydrothermal reaction. The as-synthesized CZTS nanocrystals exhibited strong light absorption over the range of wavelength beyond 1000 nm. The band gap of the material was determined to be 1.51 eV, which is optimal for application in photoelectric energy conversion device.

## Introduction

The development of new semiconductor light absorbing materials for applications in photovoltaic technologies is driven by the necessity to overcome the key issues in the current PV technologies: the high production cost of silicon wafer used in the first generation solar cells and the limited availability of raw materials such as tellurium and indium used in CdTe and Cu(Ga, In)Se_2_ (CIGS) based thin film solar cells, which has raised significant concerns over their production scale [1]. In the process of developing new PV materials that do not have the above problems, the compound Cu_2_ZnSnS_4_ (CZTS) is emerging as a promising new sustainable light absorbing material for PV technologies. As a direct band p-type semiconductor material, CZTS has a theoretical band gap of 1.5 eV and has high light absorption coefficient (>10^4^ cm^−1^) in the range of visible and near infrared irradiation of solar spectrum [2–4]. Shockley–Queisser balanced calculations have predicted that the theoretical efficiency of PVs using light absorbers like CZTS is 32% [5].

It has been proposed that high-efficiency and low-cost photovoltaic devices can be made from CZTS nanocrystals [6–7]. This is due to the fact that thin film light absorber layers with controlled thickness can be made from a slurry containing the nanocrystals by a cost-effective method such as doctor blading, spin coating and screen printing which can be scaled-up easily. The recently reported thin film solar cells based on Cu_2_ZnSn(S,Se)_4_ demonstrated a power conversion efficiency of 11.1%, which has approached the benchmark for large scale production [8]. This great achievement shows the bright future for CZTS based PVs. The highest efficiency CZTS solar cell was made using hydrazine based sol–gel method. However, hydrazine is a highly toxic, dangerously unstable solvent and requires extra caution in handling and storage [9]. Therefore, a safer, simple yet convenient method for fabrication of high quality CZTS nanocrystals is desired.

The hydrothermal method has been widely used to synthesize high quality nanocrystals with unique morphology and crystal structure due to its advantage of simplicity of the procedure and low production cost [10–17]. However, to the best of our knowledge, the formation mechanism of CZTS in the hydrothermal reaction has rarely been reported due to the complex reactions involved in the system. Herein we report the synthesis of high quality, pure kesterite phase, monodisperse CZTS nanocrystals by a one-step hydrothermal procedure. Through thoroughly investigating the factors that influence the morphology, crystal size, and growth of CZTS nanocrystals, a mechanism that depicts the formation process of CZTS compound is proposed. It is found that the tiny amount of thioglycolic acid (TGA) used in the precursor is crucial for the formation of pure kesterite CZTS nanocrystals. The roles of TGA in the hydrothermal synthesis are discussed.

## Experimental

**Materials:** All the materials were provided by Sigma Aldrich unless otherwise stated. Chemicals of copper(II) chloride dehydrate (CuCl_2_·2H_2_O), zinc chloride (ZnCl_2_) product of BDH, tin(IV) chloride pentahydrate (SnCl_4_·5H_2_O), sodium sulfide nonahydrate (Na_2_S·9H_2_O), thioglycolic acid (TGA) were all of analytical grade and used as received without further purification. Milli-Q water was used in this work.

**Synthesis of CZTS nanocrystals by hydrothermal reaction:** In a typical experimental procedure, 0.2 mmol of CuCl_2_·2H_2_O, 0.1 mmol of ZnCl_2_, 0.1 mmol of SnCl_4_·5H_2_O, 0.5 mmol of Na_2_S·9H_2_O and 18 μL of TGA were dissolved in 34 mL of Milli-Q water under vigorous magnetic stirring. The solution was then transferred to a Teflon-lined stainless steel autoclave (Parr Instrument Company) of 45 mL capacity, which was then sealed and maintained at 240 °C for 24 h. After that, the autoclave was allowed to cool to room temperature naturally. The black precipitate was collected by centrifugation and washed with deionised water and absolute ethanol for several times to remove the ions in the end product. Finally, the product was vacuum-dried at 60 °C for 5 h.

**Characterisation:** The crystallographic structure of the synthesized samples was identified by X-ray diffraction (XRD, PANanalytical XPert Pro Multi-Purpose Diffractometer (MPD), Cu Kα, λ = 0.154056 nm). The room temperature Raman spectra of the samples were recorded with a Raman spectrometer (Renishaw inVia Raman microscope). The incident laser light with the wavelength of 785 nm was employed as the excitation source in micro-Raman measurement and the spectra were collected by taking the average of 10 different spots. The quantitative elemental analysis of the samples were characterized by field emission scanning electron microscopy (FESEM, JEOL 7001F) at an acceleration voltage of 20.0 kV combined with an energy dispersive X-ray spectroscopy (EDS). Transmission electron microscopy (TEM) images of the samples were performed on a JEOL JEM-1400 microscope. High-resolution TEM (HRTEM) and selected area electron diffraction (SAED) images were obtained using JEOL JEM-2100 microscope at an accelerating voltage of 200 kV. Ultraviolet–visible (UV–vis) absorption spectrum of the sample was measured at room temperature using a Varian Cary 50 spectrometer. The chemical state of each element in the samples was determined using Kratos Axis ULTRA X-ray photoelectron spectrometer (XPS).

## Results and Discussion

### Synthesis of CZTS nanocrystals

**Figure 1:**
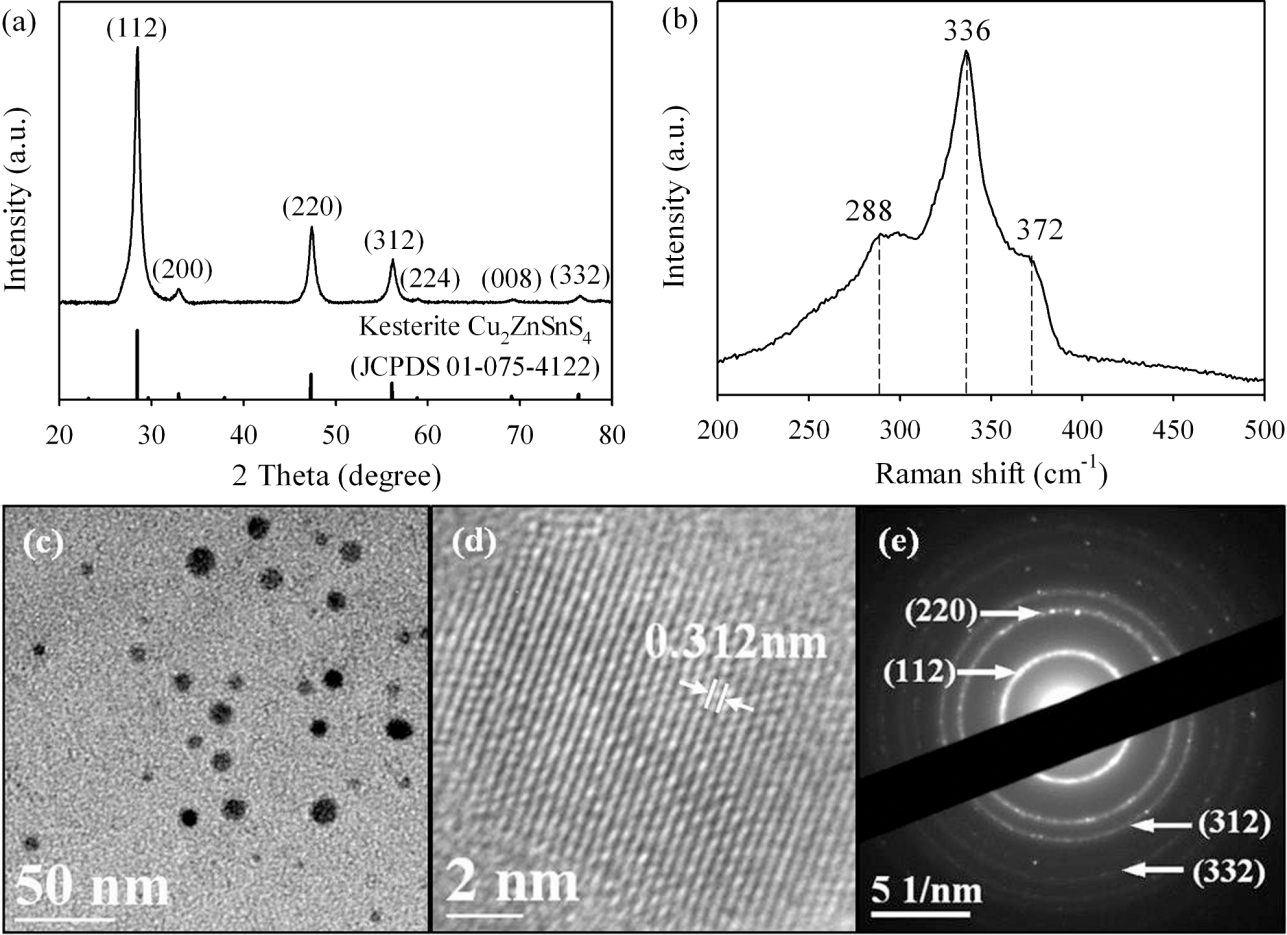
(a) XRD patterns, (b) Raman spectra, (c) TEM image, (d) HRTEM image and (e) SAED pattern of CZTS nanocrystals hydrothermally synthesized at 240 °C for 24 h.

The XRD pattern of the CZTS nanocrystals prepared at 240 °C for 24 h using 18 μL of TGA in the hydrothermal reaction is shown in Figure 1a. All the XRD diffraction peaks can be well indexed to the corresponding crystal planes of kesterite CZTS (JCPDS 01-75-4122) [5,18]. The Raman spectrum of the hydrothermal product is shown in Figure 1b. The strong peak at 336 cm^−1^ together with two shoulder peaks at 288 and 372 cm^−1^ further confirm the formation of CZTS [19]. No other characteristic peaks corresponding to impurities such as Cu_2−x_S (475 cm^−1^), SnS_2_ (315 cm^−1^), ZnS (278 and 351 cm^−1^), Cu_2_SnS_3_ (297 and 337 cm^−1^), and Cu_3_SnS_4_ (318 cm^−1^) that might form in the hydrothermal reaction are observed, suggesting the highly purity of the synthesized CZTS material [19–20]. The morphology and particle size of the CZTS nanocrystals are shown in Figure 1c, which suggests the CZTS nanocrystals are monodisperse with crystal sizes around 10 ± 3 nm. High-resolution TEM (HRTEM) image in Figure 1d illustrates the crystal interplanar spacing of 3.12 Å, which can be ascribed to the (112) plane of kesterite phase CZTS. The diffraction spots in the selected area electron diffraction (SAED) pattern illustrated in Figure 1e can all be indexed to the (112), (220), (224) and (420) planes of kesterite CZTS respectively, further confirming the phase purity of the material. The atomic ratio of Cu/Zn/Sn/S in the material is 1.97/1.04/1.03/3.96 according to energy dispersive X-ray spectroscopy (EDS) results (see Table 1), which is consistent with the stoichiometric value of 2/1/1/4 of CZTS (by considering the experimental error of EDS detector).

**Figure 2:**
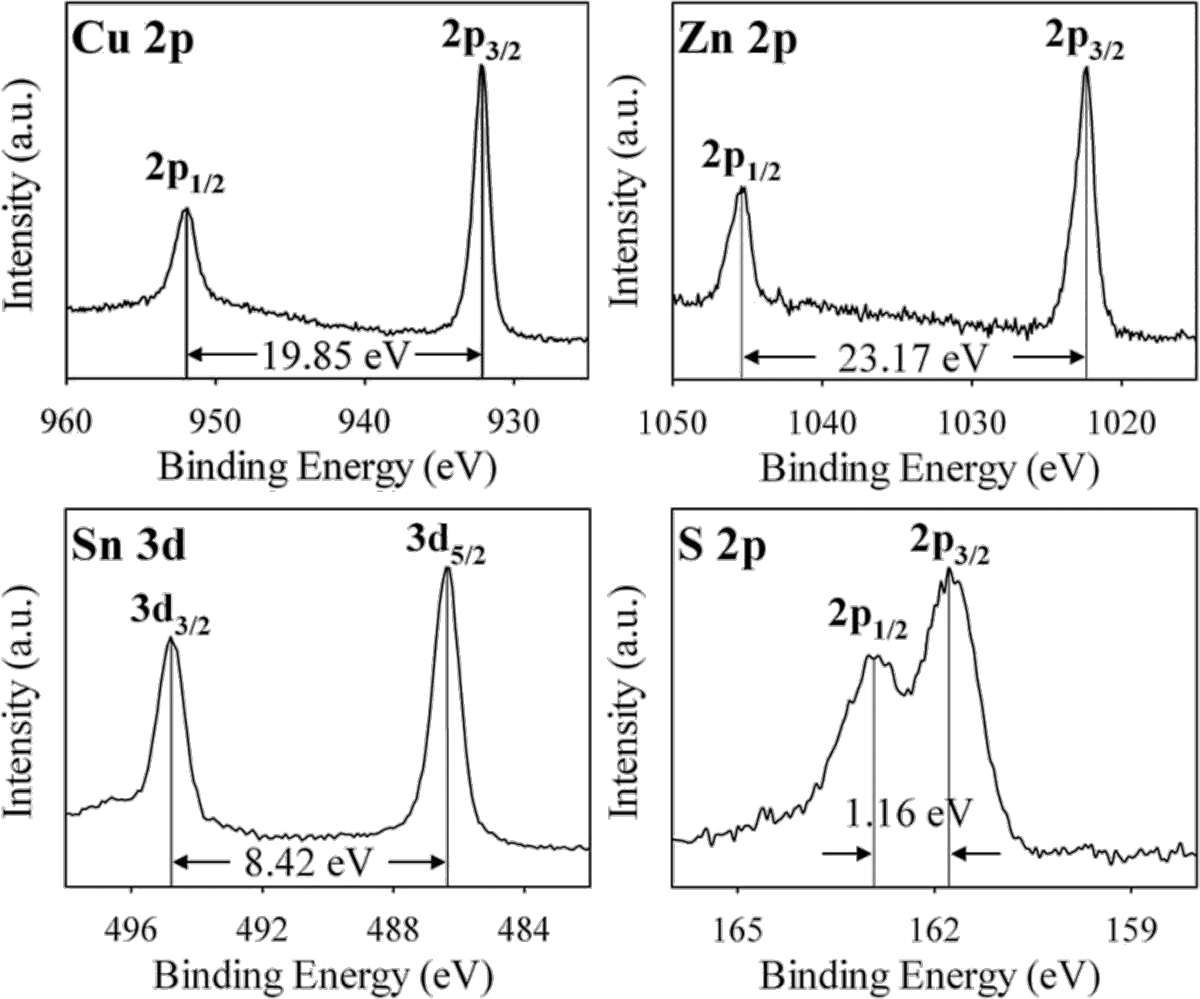
XPS spectra of CZTS nanocrystals synthesized at 240 °C for 24 h.

X-ray photoelectron spectrometry (XPS) measurement was conducted to monitor the valence states of all four elements in the as-synthesized CZTS nanocrystals. Figure 2 displays the high resolution XPS analysis for the four constituent elements: Cu 2p, Zn 2p, Sn 3d and S 2p of CZTS nanocrystals. The spectrum of Cu 2p shows two peaks at 932.14 and 951.99 eV with a splitting of 19.85 eV, which is in good agreement with the standard separation (19.9 eV) of Cu(I). The peaks of Zn 2p appear at 1022.29 and 1045.46 eV with a split orbit of 23.17 eV, which can be assigned to Zn(II). The peaks of Sn 3d show binding energies at 486.35 and at 494.77 eV respectively, which is in good agreement with the value of Sn(IV). The S 2p peaks are located at 161.76 and 162.92 eV, which are consistent with the binding energy of sulfur in sulfide state of CZTS. These results are in agreement with the reported values of the binding state of the elements of CZTS [18,21].

### Influence of different reaction condition

Different reaction conditions such as reaction temperature, reaction duration and concentration of capping agent have been reported to have significant impacts on the morphology, particle size as well as the optical properties of the materials formed in a hydrothermal reaction [17,22]. Hence, a series of experiments under different reaction conditions were carried out to understand the role of TGA and to gain in-depth insight into the formation mechanism of CZTS nanocrystals.

#### Influence of TGA concentration

**Figure 3:**
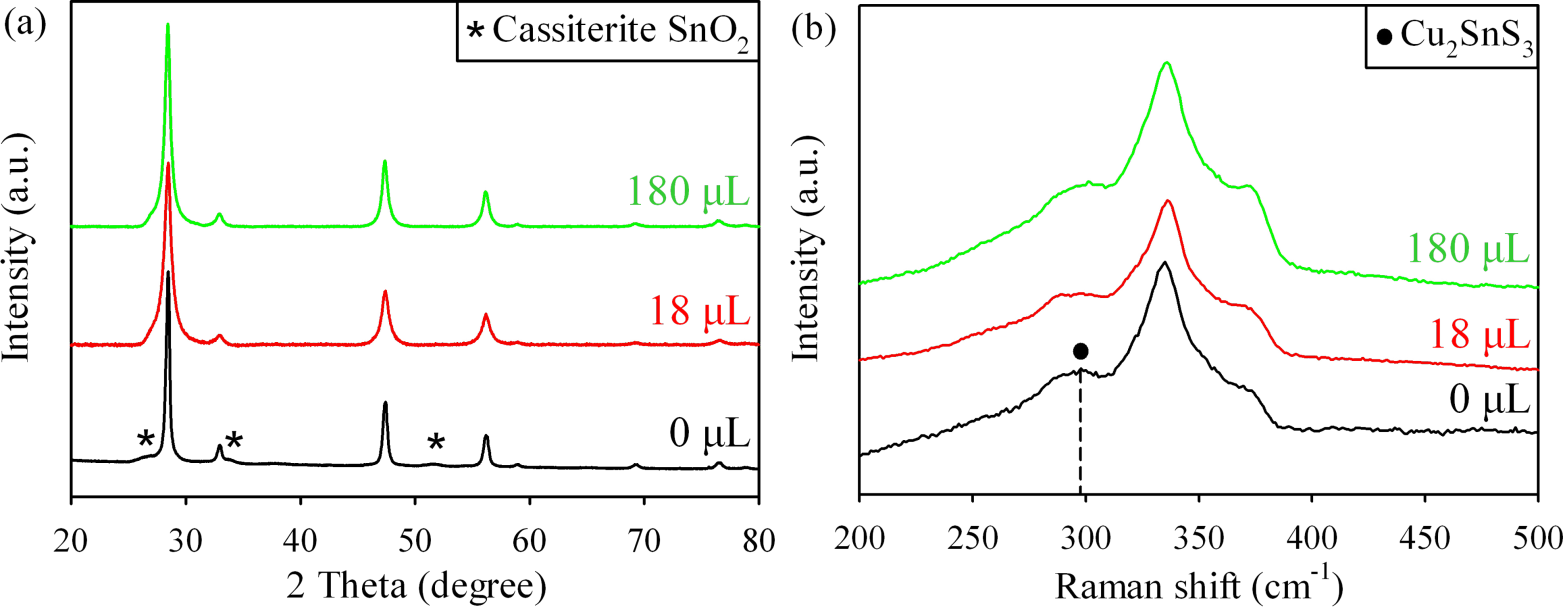
(a) XRD patterns and (b) Raman spectra of the hydrothermal products synthesized with different TGA concentration in the precursor solution.

TGA has been widely used in hydrothermal synthesis of metal sulfide such as ZnS, SnS etc. It has been reported that the content of TGA influences the morphology of the hydrothermal product [23–24]. The effect of the content of TGA on the formation of CZTS compound was investigated in this work. The XRD results of the hydrothermal products synthesized with three different TGA concentrations are shown in Figure 3a. As can be see, when there is no TGA, the XRD pattern of hydrothermal product contains the peaks corresponding to kesterite CZTS and three other peaks that are attributed to SnO_2_ (JCPDS 00-001-0625) impurity. Raman spectra in Figure 3b indicates that, in the absence of TGA, a weak peak located at 298 cm^−1^ which is assigned to ternary Cu_2_SnS_3_, is detected along with the CZTS peaks. However, the peaks corresponding to impurities disappear when tiny amount of TGA (18 μL) is added to the precursor solution. Further increase the content of TGA does not influence the XRD and Raman results of the material. Thus, the very small amount of TGA in the hydrothermal precursor solution determines the compositional purity of the synthesized CZTS material.

The quantitative elemental analysis of these three CZTS samples (Table 1) shows that the content of Sn element in the hydrothermal sample is very high ([Zn]/[Sn] = 1/1.40) without TGA. And the ratio of Cu/(Zn+Sn) and [Zn]/[Sn] is close to 1 when adding only 18 μL TGA in the hydrothermal reaction system. The Sn rich and Zn poor composition in the sample with no TGA is probably due to the formation of Cu_2_SnS_3_ and SnO_2_ impurity as confirmed by the above XRD and Raman spectrum. The EDS analysis also shows that the elemental composition of the CZTS material using excessive amount of TGA (180 μL) leads to the slightly reduced Sn content relative to Zn.

**Table 1:** Quantitative elemental analysis of CZTS nanocrystals synthesized with different TGA content in the precursor solution of the hydrothermal reaction.

Ratio	0 μL	18 μL	180 μL

Cu/Zn/Sn/S	2.02/0.84/1.18/3.96	1.97/1.04/1.03/3.96	1.96/1.02/0.93/4.09
[Cu]/([Zn+Sn])	1/1.00	1/1.05	1/0.99
[Zn]/[Sn]	1/1.40	1/0.99	1/0.91

**Figure 4:**
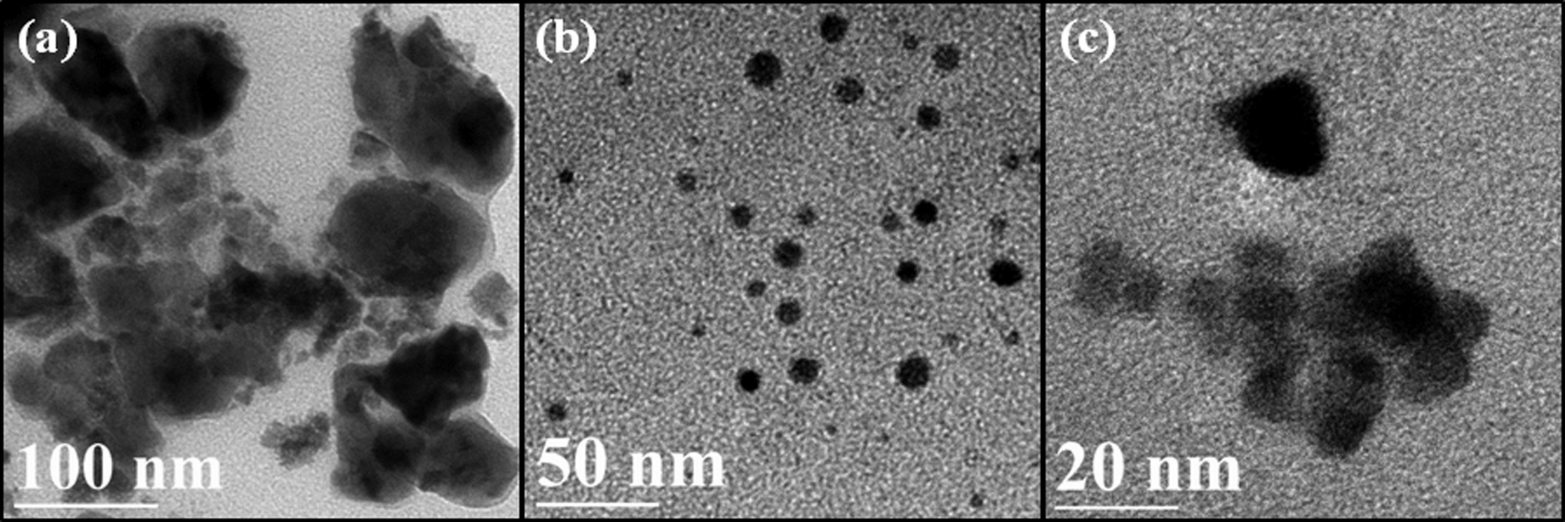
TEM images of CZTS nanocrystals synthesized using (a) 0, (b) 18 and (c) 180 μL of TGA at 240 °C for 24 h.

The morphology of the synthesized CZTS nanocrystals prepared with the three different amounts of TGA measured by TEM is shown in Figure 4. It shows that when there is no TGA in the precursor solution, agglomerates with irregular shape with size ranging from 10–150 nm are obtained. When 18 μL TGA is used in the reaction system, uniform and monodisperse CZTS nanocrystals with an average size of 10 nm are obtained. With increasing the TGA concentration to 180 μL, the size distribution of the CZTS nanocrystals becomes less uniform and some triangular-like shape nanocrystals are observed. The above results demonstrate that high concentration of TGA is not favourable for the formation of monodisperse CZTS nanocrystals. At a high concentration, TGA might form a colloid which wraps a certain surface of CZTS particles, inhibiting the growth of crystals in all directions [25]. Hence, it is rational to conjecture that TGA might play two key roles in this work. One is to prevent aggregation of CZTS nanocrystals by capping on the generated nanocrystals to reduce the surface energy (steric hindrance) during the hydrothermal process; the other role is selective adsorption on certain facets of CZTS nanocrystals and kinetic control of the growth rates of these facets [4,25].

#### Influence of reaction duration

**Figure 5:**
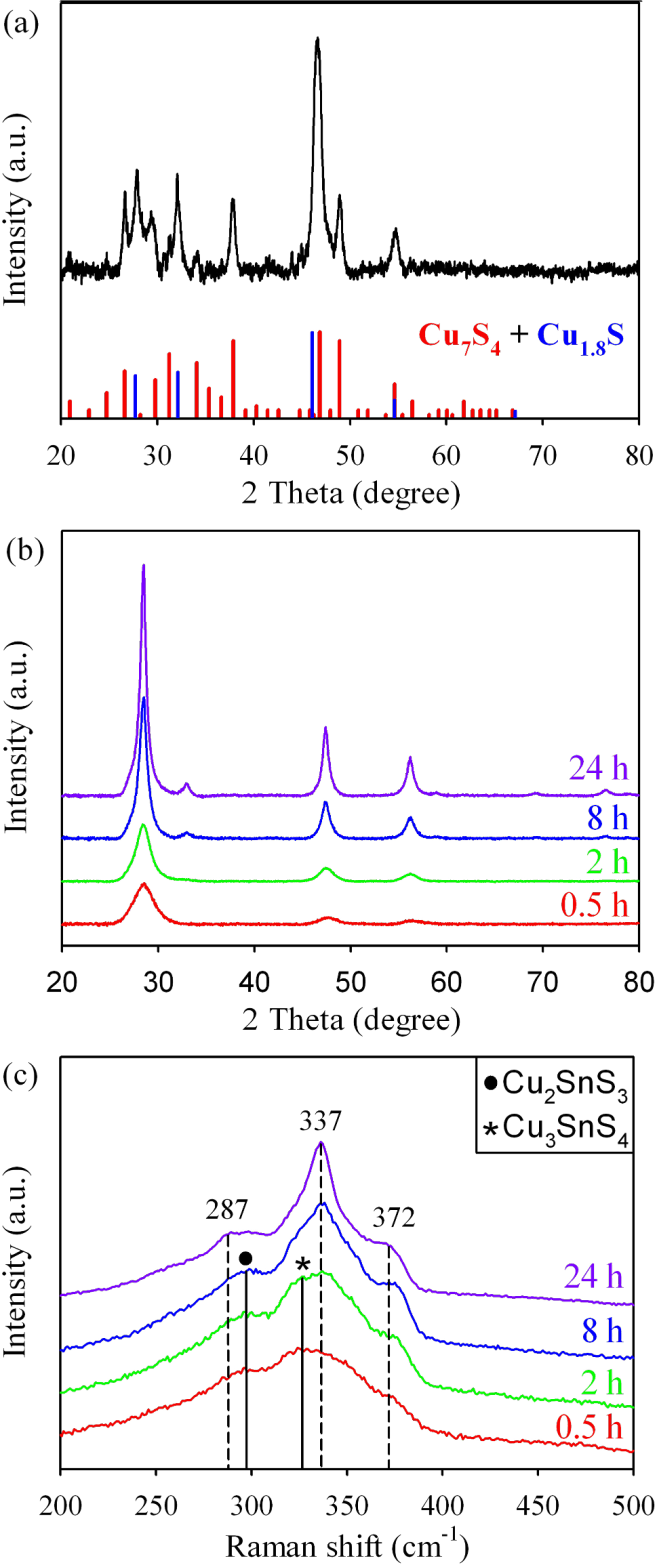
(a) XRD of the precipitate collected from the precursor solution prior to hydrothermal reaction and (b) XRD patterns and (c) Raman spectra of the samples synthesized at different reaction duration.

Figure 5b,c shows the XRD patterns and Raman spectra of the CZTS nanocrystals synthesized at different hydrothermal reaction duration (from 0.5 h to 24 h). The XRD pattern of the precipitate collected from the hydrothermal precursor solution prior to the reaction is shown in Figure 5a. The result suggests that Cu_7_S_4_ (JCPDS 23-0958) and Cu_1.8_S (JCPDS 56-1256) are formed immediately in the precursor solution prior to the hydrothermal reaction. When the hydrothermal reaction is proceeded for only 0.5 h (Figure 5b), three XRD peaks that can be assigned to kesterite CZTS are observed. The intensity of the diffraction peaks increases gradually with the increase of the reaction time. A characteristic peak located at around 32.9° corresponding to the (200) plane of kesterite CZTS is noticeable only when the reaction duration is extended to 8 h and beyond. The gradual increment of the intensity of diffraction peaks suggests the improvement of crystallinity of the CZTS nanocrystals. However, the Raman spectra (Figure 5c) of the hydrothermal products synthesized at different reaction duration show that, at a shorter reaction time (less than 8 h), the hydrothermal products contain a mixture of CZTS, Cu_2_SnS_3_ and Cu_3_SnS_4_. Pure phase CZTS nanocrystals are only obtained at reaction duration of 24 h. The Raman spectra also show that, the intensity of CZTS peak at 337 cm^−1^ increases and the peak at 327 cm^−1^ which belongs to Cu_3_SnS_4_ decreases as the reaction time is prolonged [26]. The red shift of the peak at 298 to 287 cm^−1^ denotes the complete transformation of Cu_2_SnS_3_ to CZTS at 24 h hydrothermal reaction. Since no peak corresponding to ZnS is observed in the entire Raman spectra, it suggests that CZTS compound in the hydrothermal reaction might be formed through diffusion of Zn ion to the ternary Cu*_x_*SnS*_y_* (*x* = 2,3, *y* = 3,4) compound.

The atomic ratios of [Cu]/([Zn]+[Sn]) and [Zn]/[Sn] of the synthesized hydrothermal products obtained at different reaction duration are shown in Table 2. The much higher content of copper in the sample obtained prior to the hydrothermal reaction and the nearly two-fold of Cu relative to S is consistent with the observation of Cu_2−_*_x_*S product by the XRD measurement as discussed above. As the reaction time is prolonged from 0.5 h to 24 h, the [Cu]/([Zn]+[Sn]) ratio is reduced from 1/0.82 to 1/1.05, while the [Zn]/[Sn] ratio shows a little change from 1/0.93 to 1/0.99. The nearly stoichiometric composition (1.97/1.04/1.03/3.96) of CZTS is obtained at a reaction time of 24 h. Since no ZnS is detected in the samples synthesized at 0.5 and 2 h, we believe that the content of Cu_2_SnS_3_ impurity in these hydrothermal products is very low. The high content of Zn and Sn compound at 0.5 h and 2 h also suggests that CZTS compound is formed rapidly in the hydrothermal reaction.

**Table 2:** Quantitative elemental analysis of CZTS nanocrystals synthesized at different reaction duration.

Ratio	0 h	0.5 h	2 h	8 h	24 h

Cu/Zn/Sn/S	5.0/0.1/0.1/2.8	2.1/0.9/0.8/4.1	2.1/1.0/0.9/4.0	2.1/1.0/1.0/3.9	2.0/1.0/1.0/4.0
[Cu]/([Zn+Sn])	1/0.04	1/0.82	1/0.90	1/0.97	1/1.05
[Zn]/[Sn]	—	1/0.93	1/0.92	1/0.96	1/0.99

**Figure 6:**
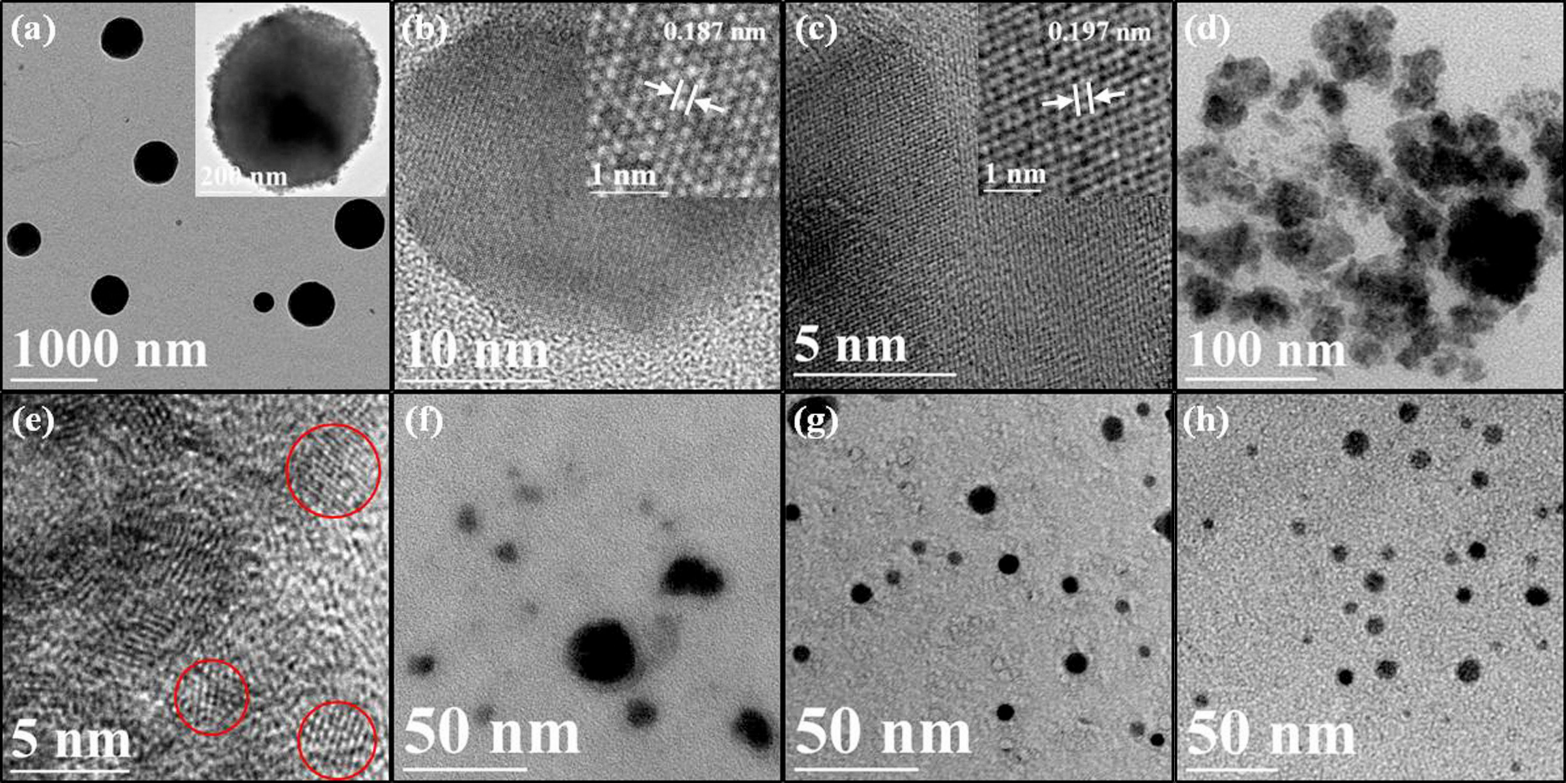
TEM images of (a, b, c) Cu_7_S_4_ and Cu_1.8_S nanocrystals collected prior to hydrothermal reaction and CZTS nanocrystals synthesized at different reaction duration: (d, e) 0.5 h, (f) 2 h, (g) 8 h, and (h) 24 h respectively.

The TEM images of the hydrothermal products collected at different reaction duration are shown in Figure 6. Figure 6a illustrates that, prior to the hydrothermal process, the precipitate obtained from the precursor solution is consisting of microspheres with size around 20–250 nm. The HRTEM indicates that the microparticle is the result of aggregation of numerous oval-like nanocrystals with size ranging from 10–30 nm. The HRTEM image of the material (inset of Figure 6b) shows the lattice fringe of a nanocrystal with an interplanar spacing of 1.87 Å, which is in good agreement with the (886) plane of monoclinic structure of Cu_7_S_4_. Besides that, the lattice fringe of nanocrystal with an interplanar spacing of 1.97 Å which can be ascribed to (220) plane of cubic structure of Cu_1.8_S was also found as illustrated in the inset of Figure 6c. These finding are consisting with the above shown XRD pattern. Figure 6d and 6e show that when the hydrothermal reaction is proceeded for 0.5 h, the dominant products are nanocrystals with irregular size of about 2–5 nm. The interplanar spacing of the crystals is 3.125 Å, which belongs to the (112) plane of CZTS. This result further confirms the rapid formation of CZTS compound in the hydrothermal reaction. Figure 6f shows that after hydrothermal reaction for 2 h, the agglomeration starts to break into small particles with size ranging from 3–20 nm. As the reaction time is prolonged to 8 h (Figure 6g), nanocrystals with size in the range of 5–10 nm appear in the product, and meanwhile the large agglomerates disappear. Upon gradual evolution of the CZTS nanostructures, nanoparticles with uniform distribution are obtained after reaction duration of 24 h (Figure 6h).

#### Formation mechanism

**Figure 7:**
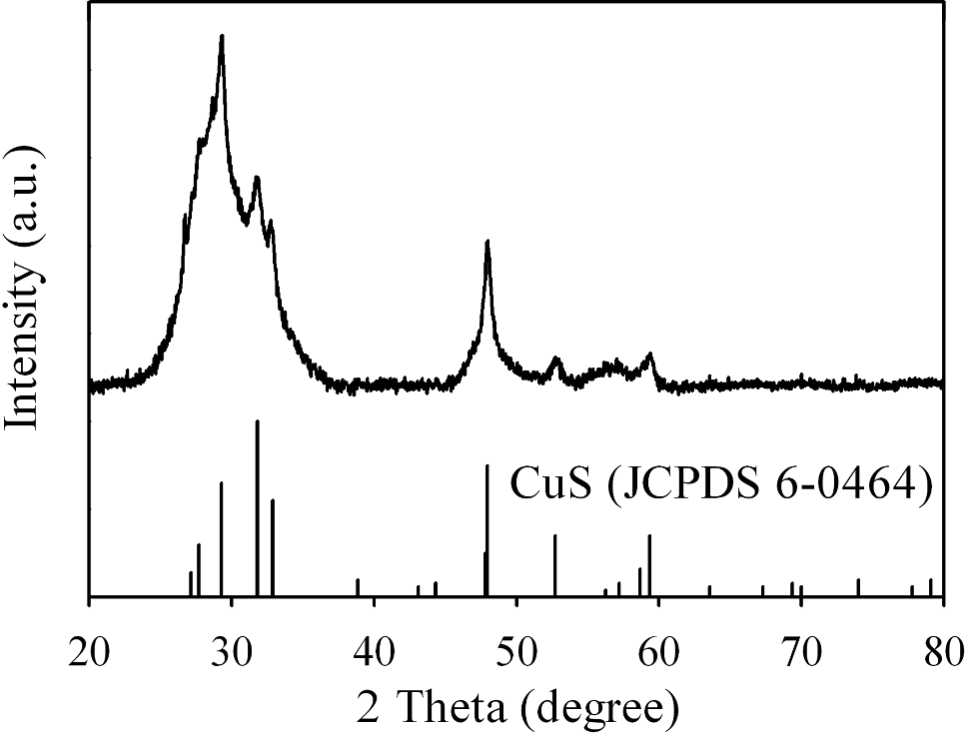
XRD pattern of the precipitate collected from the precursor solution without TGA prior to hydrothermal reaction.

It is normally assumed that metal cations in a hydrothermal reaction are firstly associated with TGA in the precursor solution to form metal-TGA complexes prior to the hydrothermal reaction [22–23]. However, the formation of Cu_7_S_4_ and Cu_1.8_S compounds in our case suggests that Cu^2+^ is reduced to Cu^+^ by interaction with the –SH (thiol) group of TGA (oxidation of TGA to dithiodiglycolate) [27]. The XRD pattern of the precipitate collected from the precursor solution without TGA prior to hydrothermal reaction reveals that CuS (JCPDS 6-0464) instead of Cu_2−_*_x_*S (*x* = 0–0.2) is formed (Figure 7). This confirms the reduction role played by TGA in the hydrothermal reaction system.

**Figure 8:**
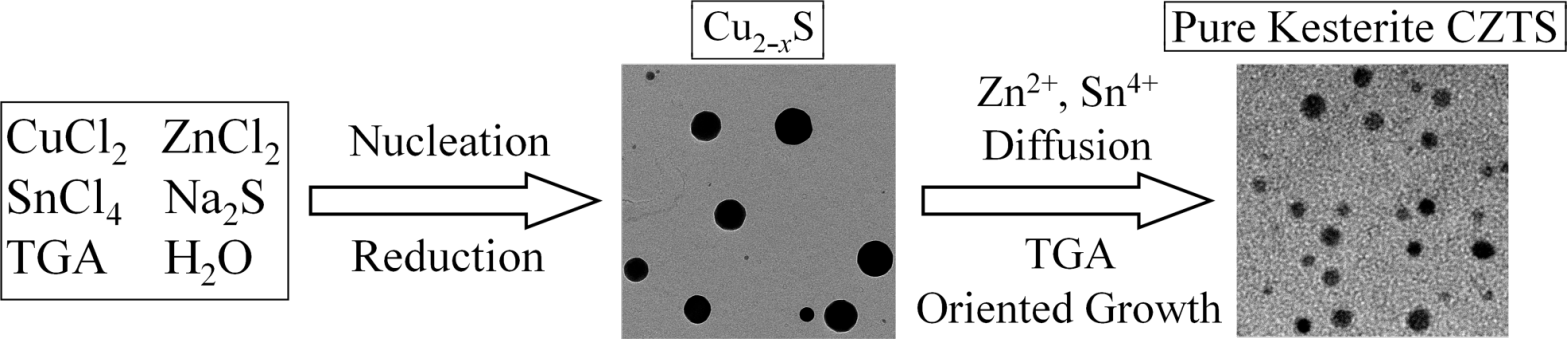
Schematic illustrations of the formation process for kesterite CZTS nanoparticles.

We believe that the initially formed Cu_2−_*_x_*S nanocrystals in the precursor solution prior to hydrothermal reaction act as nuclei for the formation of kesterite CZTS. The formed Cu_7_S_4_ and Cu_1.8_S material has a monoclinic and cubic crystal structure, respectively as confirmed by XRD measurement shown in Figure 5a. At relatively high reaction temperature, the copper ions in Cu_2−_*_x_*S have a relatively high mobility which can accommodate an exchange with other metal ions at a low energy cost [28]. In addition, the crystal structure of Cu_1.8_S has a cubic close packing (ccp) array of sulfur ions which is similar to the arrangement of sulfur in kesterite CZTS crystal framework. Hence, the interdiffusion of cations such as Zn^2+^ and Sn^4+^ to Cu_2−_*_x_*S crystal to form CZTS compound is feasible because there is little lattice distortion in such process [29]. Thus, the typical reaction condition (220 °C and above) is believed to facilitate the chemical transformation from Cu_2−_*_x_*S to CZTS. Since no other binary products such as SnS_2_ and ZnS are discovered in the entire hydrothermal process, and only Cu_2_SnS_3_ and Cu_3_SnS_4_ are detected in the Raman spectra, we believe Sn^4+^ cations are firstly incorporated into the crystal lattice of Cu_2−_*_x_*S and replaced parts of Cu^+^ ion, followed by the rapid doping of Zn^2+^ to form CZTS compound in the hydrothermal process. The formation of CZTS compound as confirmed by TEM at the very short reaction time (0.5 h) suggests the fast diffusion rate of Zn and Sn ions to the lattice of Cu_2−_*_x_*S nuclei crystals in the hydrothermal process. Moreover, when the reaction duration is extended to 8 h, the Zn/Sn ratio is increased to 1/1.04 which matches well with the theoretical value of 1:1 in CZTS. With the proceeding of the reaction, the primary CZTS crystal nucleus grows to nanoparticles with different sizes. Based on Ostwald ripening process, the small crystal nucleus will grow up to form larger crystals because the large one has lower surface free energy [30]. TGA molecules which are adsorbed on the nanocrystals surface may restrict the growth of CZTS crystals and slow down the growth process, leading to the formation of monodisperse CZTS nanocrystals. Based on the above analysis, a schematics showing the formation mechanism for CZTS compound in the hydrothermal reaction is shown in Figure 8.

**Figure 9:**
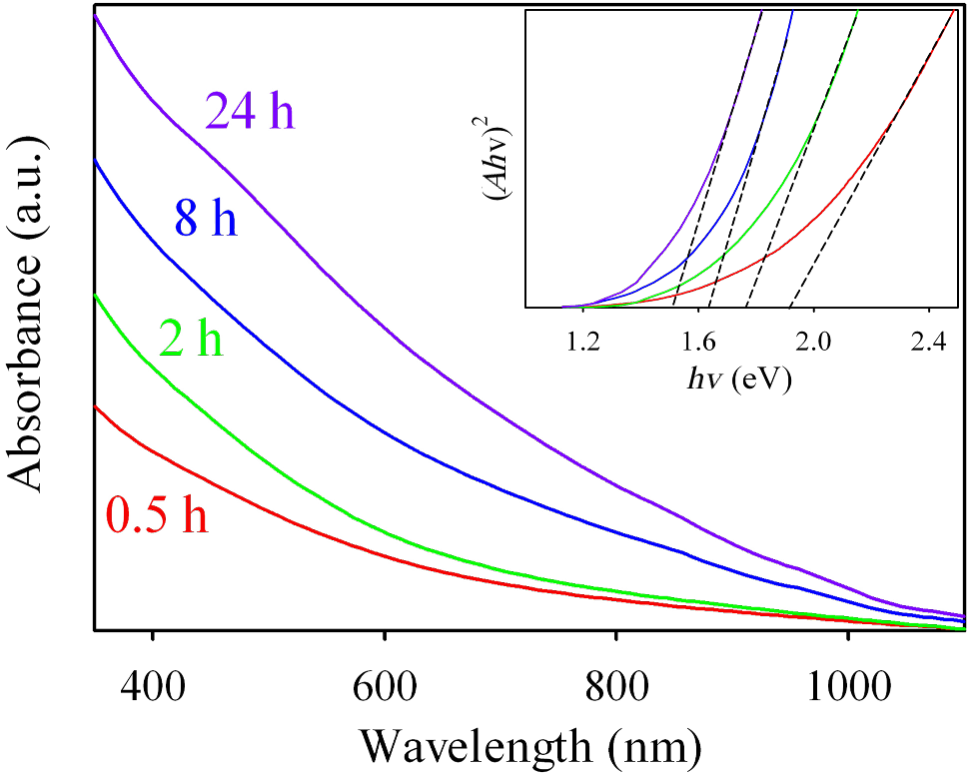
UV–visible absorption spectra of the CZTS nanocrystals synthesized at different reaction duration and the inset image shows the (*Ah*ν)^2^ vs *h*ν plots with corresponding fitting of the samples.

The UV–visible spectra of the hydrothermal samples synthesized at different reaction time are shown in Figure 9. It is found that the onset for light absorption of the material gradually shifts to longer wavelengths with the elongation of the reaction duration. The calculation of the band gap of the materials which is determined by extrapolation of the plot of (*Ah*ν)^2^ vs *h*ν (Inset of Figure 9) where *A* = absorbance, *h* = Planck’s constant, and ν = frequency, shows that the hydrothermal products at reaction time of 0.5, 2, 8, and 24 h have band gap of 1.92, 1.76, 1.63 and 1.51 eV respectively. The decrement of the band gap value is due to the improvement of CZTS purity because impurities such as Cu_2_SnS_3_ and Cu_3_SnS_4_ have larger band gap than CZTS [31–32].

## Conclusion

High quality, pure kesterite phase CZTS nanocrystals with uniform size distribution have been successfully synthesized by a facile one-step hydrothermal route based on a precursor solution containing thioglycolic acid (TGA) as surfactant. The role of TGA in the hydrothermal reaction is clarified and a formation mechanism of CZTS compound in the hydrothermal reaction is proposed. It is believed that the formation of CZTS is initiated by the formation of Cu_2−_*_x_*S nanocrystals as a result of reduction of Cu^2+^ by TGA to become Cu^+^. This is followed by the rapid diffusion of cations Sn^4+^ and Zn^2+^ to the crystal framework of Cu_2−_*_x_*S to form CZTS. The good optical properties and suitable band gap of 1.51 eV of the synthesized CZTS nanocrystals indicate the promise of this material for application in low cost thin film solar cells.
